# Efficacy of neuromobilization in the treatment of low back pain: Systematic review and meta-analysis

**DOI:** 10.1371/journal.pone.0302930

**Published:** 2024-05-07

**Authors:** Quanzheng Chen, Zhenshan Wang, Xian Chen, Jinchao Du, Shuna Zhang

**Affiliations:** 1 Department of Physical Education and Health, Guangxi Normal University, Guilin, China; 2 Department of Rehabilitation Medicine, Shandong Second Medical University, Weifang, China; King Khalid University, SAUDI ARABIA

## Abstract

**Background:**

Low back pain (LBP) is a leading cause of disability. Neuromobilization (NM) as a physical therapy technique, offers some degree of symptom improvement. However, some studies have shown that NM can significantly reduce the symptoms of LBP, while others have failed to find similar positive effects.

**Objective:**

This study aims to investigate the effectiveness of NM for LBP.

**Data sources:**

A literature search was conducted across five databases (MEDLINE, Embase, Cochrane Library, PubMed, and Web of Science) from their inception to December 2023. Study main measures assessed pain, disability, and straight leg raise angle to determine the degree of improvement in patients.

**Results:**

Seven randomized controlled trials were included in the analysis. The findings indicated that NM interventions in patients with LBP were more effective than control groups in improving Visual Analog Scale scores (mean difference = 0.62, 95% CI (0.03, 1.21)) and Oswestry Disability Index scores (mean difference = 7.54, 95% CI (4.98, 10.10)). There was no significant difference in straight leg raise results (mean difference = 0.18, 95% CI (-0.08, 0.44)).

**Conclusions:**

NM demonstrated effectiveness in improving Visual Analog Scale and Oswestry Disability Index outcomes in patients with LBP, but straight leg raise outcomes are still uncertain and until more high-quality studies are included, the effectiveness of NM for SLR remains unknown.

## Introduction

Low back pain (LBP), one of the main causes of disability [1], can be attributed to a variety of factors, including psychological and physical [2]. LBP is typically associated with degenerative lesions caused by various factors, such as sprains, sciatica, scoliosis, slipped discs, and radiculopathy and so forth [3]. Not only does it put economic pressure on the patients themselves [4], but it also adds a potential burden on society [5]. Research findings suggest that a substantial proportion of the adult population, potentially up to 84%, have encountered episodes of LBP at some point in their lifespan. Furthermore, these investigations elucidate a tendency for the expenditure on LBP treatment to increase progressively over time [6].

Exercise is often considered the best treatment for LBP [7]. A study documented the establishment of the Chronic Pain Management Guidelines Committee, tasked with addressing chronic pain. The committee observed that the potential adverse effects associated with the utilization of opioids and cannabinoids might surpass the therapeutic advantages across a majority, if not all, of the examined conditions. Consequently, pharmacotherapy might not represent the optimal approach. Furthermore, the committee advocates for exercise as the primary treatment in the management of chronic osteoarthritis and low back pain [8]. However, it is worth noting that medication is currently the preferred option for doctors [9]. Although medication, exercise therapy, and physical factor therapy are commonly used in clinical practice [10], there is an ongoing search for a more cost-effective treatment for LBP. Researchers have found that neuromobilization (NM) shows promise in relieving LBP [11]. NM is an intervention that aims to restore homeostasis of the nervous system or its surrounding structures through manipulation or exercise [12], it involves various techniques, including tension techniques and slider techniques [13]. Several studies have shown that the use of NM in the treatment of neurological diseases can improve joint range of motion better than other techniques in the long term [14]. NM can be applied to adjacent structures, and may through mechanical mechanism to improve symptoms including relieve pain, improve the disabled, reduce edema, and so forth [12].

Although NM is considered an effective treatment for LBP, previous clinical studies have shown inconsistent results. Some studies have shown that NM can significantly reduce the symptoms of LBP [15, 16], while others have failed to find similar positive effects [17]. Therefore, the main objective of this study is to analyze the existing evidence on the effectiveness of NM for the improvement of the syndromes (pain, disability, etc.) of LBP.

## 1 Materials and methods

### 1.1 Literature search strategy

This study has been registered with PROSPERO (CRD42023414200). Two researchers independently searched for randomized controlled trial articles published in the following databases: MEDLINE, Embase, Cochrane Library, PubMed, and Web of science [18]. Used 3 sets of keywords: (1) Random, control, Randomized control; (2) neuromobilization, Nerve mobilization, neural mobilization, NM; (3) Mechanical Low Back Pain, Lower back pain, Lumbago, LBP, low back pain. The search period and scope of this study included the period from the creation of the database until December 8, 2023. An example of the search in PubMed can be seen in supplementary material (S1 Checklist).

### 1.2 Inclusion and exclusion criteria

#### 1.2.1 Inclusion criteria

The literature cited includes randomized controlled trials (RCTS). The subjects were LBP patients who received NM or NM combined with other treatment techniques. In addition, the literature included in this study should be in English. Evaluation measures for inclusion should include the Visual Analogue Scale (VAS) for pain intensity assessment or the Oswestry Disability Index (ODI) for functional impairment or the Straight Leg Raised (SLR) test for lumbar mobility and potential nerve root involvement.

VAS scale is a scale used to assess pain, usually on a scale of 1–10, with a lower score indicating less pain intensity, and conversely, a higher score indicating greater pain intensity [19]; ODI scale is a scale used to assess dysfunction and typically has 10 questions, each corresponding to a score of 0–5 on a 50-point scale, with a lower score representing better functioning and, conversely, a higher score representing a higher degree of dysfunction [20]. The straight leg raise is often used to check the patient’s sciatic nerve, when patients supine leg lifts, along with the rising of the leg Angle, such as patients with sciatic nerve problems, can appear the numbing pain (usually within 70°) [21].

#### 1.2.2 Exclusion criteria

The criteria for excluding articles were non-English language, or the experimental data were not available.

### 1.3 Literature screening and data extraction

The literature retrieved from the database was amassed by two researchers employing EndNote X9 software. Initially, a process of duplicate identification was undertaken, followed by a comprehensive screening to ascertain the inclusion of pertinent literature. The final phase entailed meticulous data extraction from the selected literature, encompassing key details such as authorship, publication date, gender distribution, sample size, interventions employed, duration of interventions, and evaluation criteria. In the whole process, there are X.C, Z.W involved, if there is any disagreement about the same literature, the Q.C researcher acts as a mediator and makes the final decision [22].

### 1.4 Study quality evaluation and risk of bias assessment

The screened literature underwent rigorous assessment for quality and bias utilizing the Cochrane Library handbook tool. Six key domains were scrutinized, encompassing the random assignment sequence, concealed assignment scheme, blinding procedures, management of incomplete outcome data, selective reporting of results, and identification of any other potential biases, evaluation process is consistent with 1.3 [2].

### 1.5 Missing data processing

Incomplete or missing data within the literature significantly influenced research outcomes, underscoring the pivotal role of maintaining data integrity to enhance the reliability of research findings. In instances where data were deficient or incomplete, researchers prioritized strategies to mitigate these limitations. This involved potential outreach to authors via email or telephone during the data extraction phase to procure the absent information. However, in cases where contact with authors proved unfeasible or relevant data remained inaccessible, alternative methodologies were implemented. For instance, when standard deviations were absent from the literature, researchers resorted to utilizing analogous standard deviations from comparable studies or employed statistical methodologies to infer or interpolate missing values [23].

### 1.6 Bias report

If the number of included articles exceeded 10, publication bias would have been assessed using a funnel plot [24].

### 1.7 Data analysis

The data extracted from the encompassed literature underwent statistical scrutiny employing RveMan 5.4, a software resource facilitated by the Cochrane website. For continuous variables, statistical analyses were executed utilizing the mean difference (MD) alongside its corresponding 95% confidence interval (CI). For MD and standard deviation (SD) data extraction, this study was guided by the Cochrane Handbook of Systematic Reviews [25]. Heterogeneity was evaluated through the I^2^ statistic and chi-squared tests. Elevated I^2^ values suggested considerable heterogeneity across the included studies. Typically, if the number of studies exceeded five (n>5) and the I^2^ value surpassed 50%, a random-effects model was selected for the analysis. Conversely, when the number of studies amounted to five or fewer (n≤5), a fixed-effects model was applied. The statistical significance of the final outcomes was determined if the p-value fell below 0.05. However, in scenarios characterized by statistical heterogeneity between effect sizes, the appropriateness of the fixed-effect model was deemed inadequate. In such instances, where true homogeneity could not be presumed, the consideration of a random-effects model was warranted [26, 27]. In Meta analysis, if the number is too little, it is difficult to find the reasons of the asymmetric. Because too little number included in this study (< 10), so there is no bias assessment.

## 2 Results

### 2.1 Search result

**Fig 1:**
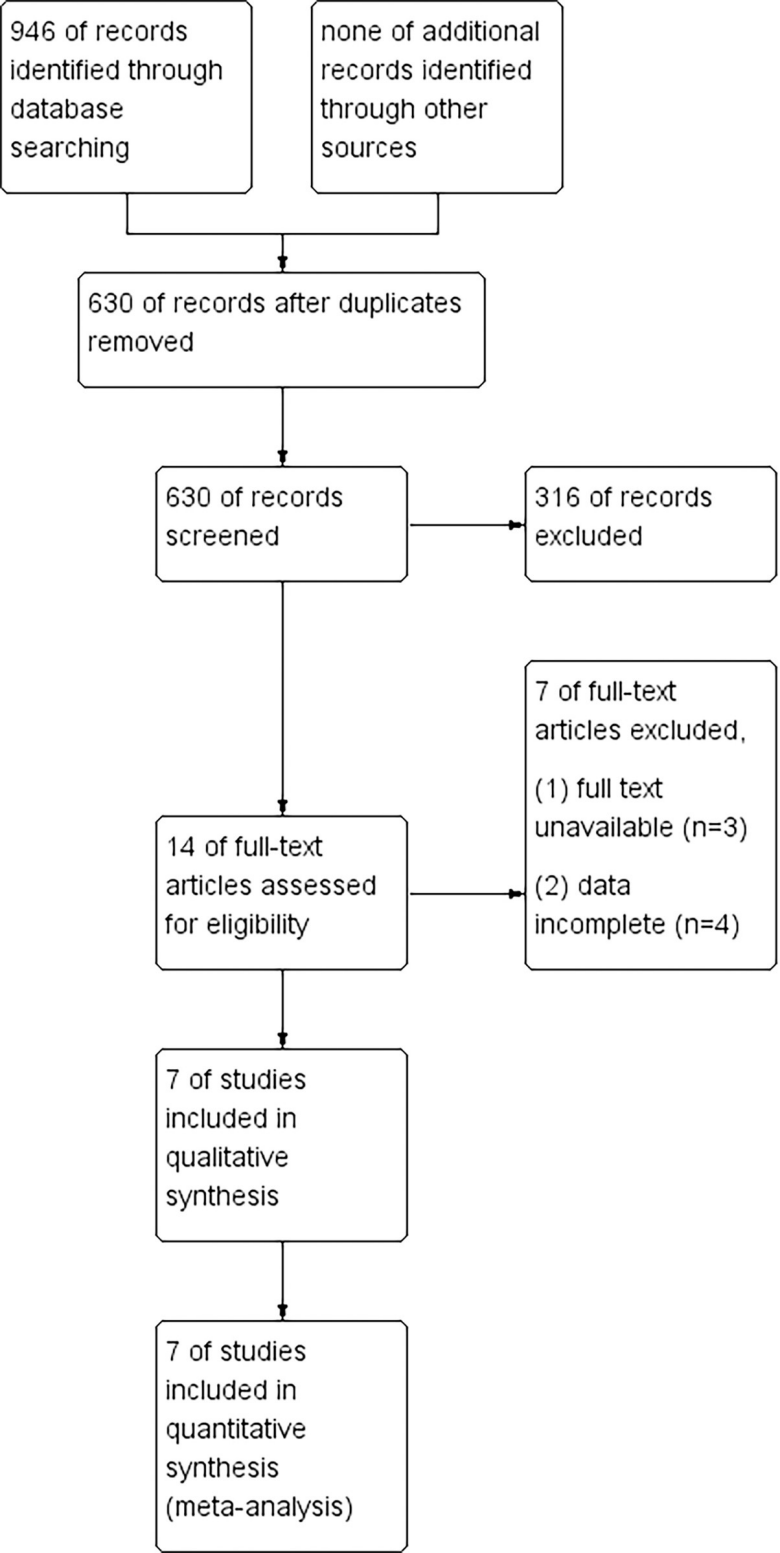
Flow chart. Note: MEDLINE (99), PubMed (139), Web of science (254), Cochrane (264), Embase (190).

As depicted in Fig 1, the initial stage of the study involved retrieving a total of 946 articles through the database search, with no additional literature acquired through alternative channels. Following this, a rigorous process of duplicate identification led to the removal of 316 redundant articles, thereby yielding a corpus of 630 unique articles. Subsequent screening of titles and abstracts resulted in the selection of 14 papers based on predefined inclusion and exclusion criteria. These selected papers underwent thorough evaluation through examination of their full text, culminating in the inclusion and analysis of 7 papers for data extraction purposes.

### 2.2 Basic characteristics of the literature

Seven papers were screened according to the inclusion and exclusion criteria [17, 28–33], a total of 288 subjects were included, and the publication period of the included literature was 2012–2022. In the study, the experimental group received NM and combined protocols as the primary intervention. On the other hand, the control group was treated with either conventional treatment protocols or a placebo intervention. The basic information of literature is shown in **Table 1**.

**Table 1:** Basic information.

Study	sample size E/C	age	Intervention		Time to intervention	outcome measure
			experiment group	control group		
Muhammad Adnan 2022	-	-	conventional treatment+NM	conventional treatment+Bent Leg Raise	4 weeks	NPRS、ODI、ROM
Alvaro ’Cu ~ nado Gonz’ alez 2021	E: 13/13 C: 7/18	E: 42.7±13.6 C: 44.6±11.3	NM	shame NM	1 weeks	VAS、SLR
Myoung-Kwon Kim 2012	E: 5/7 C: 7/5	E: 36.5±10.6 C: 59.9±10.4	NM+Electromagnetic Diathermy	NM	-	ODI、LOS
Vedat Kurt 2020	-	E: 39.45 ± 8.55 C: 38.33 ± 9.70	ChattanoogaTM ultrasound+local hot pack+ChattanoogaTM TENS+NM	ChattanoogaTM ultrasound+local hot pack+ChattanoogaTM TENS	3 weeks	VAS、SLR、ODI
Gustavo Plaza-Manzano 2019	E: 8/8 C: 8/8	E: 47.0±8.0 C: 45.5 ± 6.0	Motor Control + Neurodynamic	Motor Control	2 months	NPRS、S-LANSS、RMDQ、SLR、PPT
Neha Tambekar 2016	E: 8/7 C: 8/8	E: 32.26±4.81 C: 34.06±8.28	Butler’s neural tissue mobilization technique	Mulligan bent leg raise technique	once	VAS、SLR
ZAINAB 2022	E: 30/10 C: 28/9	E: 39.42±7.62 C: 38.13±8.03	sciatic nerve mobilization+conventional treatment	conventional treatment	2 weeks	SLR、NPRS、MODI

Notes: NM: Neuromobilization; NPRS: Numeric pain rating scale; ODI: Oswestry disability index; ROM: Range of motion; LOS: Location of symptoms scale; S-LANSS: Self-report Leeds Assessment of Neuropathic Symptoms and Signs Scale; RMDQ: Roland-Morris Disability Questionnaire; PPT: Pressure pain threshold; MODI: Modified Oswestry disability index; VAS: visual analog scale; "-" indicates not clarity.

### 2.3 Evaluation of the quality of the included literature and risk bias assessment

**Fig 2:**
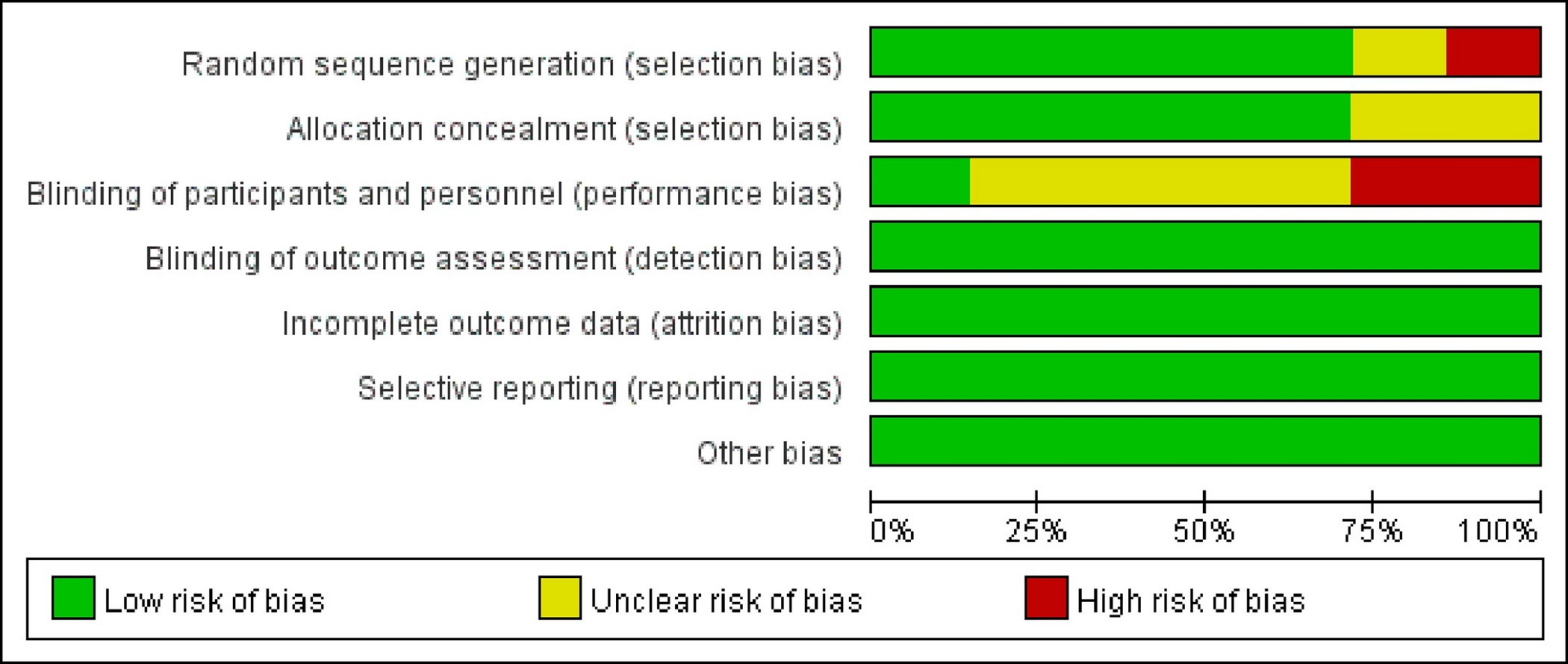
Quality and risk bias assessment.

Quality and risk of bias were meticulously evaluated for the seven included articles based on Cochrane criteria. The assessment revealed that the majority of the studies demonstrated satisfactory results in terms of bias, with most indicating low risk across various domains such as random assignment sequence, concealed assignment scheme, incomplete outcome data, selective reporting of results, and identification of potential biases. However, shortcomings were observed, particularly in the domain of blinding. Notably, two studies lacked pertinent information regarding blinding procedures, suggesting a potential high risk of bias and relatively lower quality. Additionally, four studies were noted to involve either single blinding or unclear reporting in their narratives, thereby warranting a medium quality rating. Furthermore, in the section pertaining to randomization, one study failed to specify the method of assignment, indicating a higher risk of bias and diminished quality. These findings are visually depicted in **Fig 2**.

### 2.4 Meta-analysis results

#### 2.4.1 Visual analog scale score

**Fig 3:**
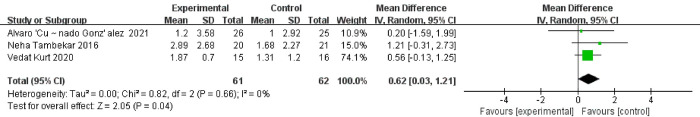
Comparison of VAS scores between experimental and control groups.

Three studies reported effects of NM on pain through the use of VAS [17, 29, 31]. The test of heterogeneity yielded the following results: χ^2^ = 0.82, I^2^ = 0%, P = 0.66, using a fixed-effects model. The meta-analysis results demonstrated a mean difference (MD) of 0.62, with a 95% confidence interval (CI) of (0.03, 1.21), and a corresponding p-value of 0.04. These findings indicate statistical significance and suggest that the improvement in VAS scores with NM was superior to that of the control group. **Fig 3**.

#### 2.4.2 Oswestry disability index score

**Fig 4:**
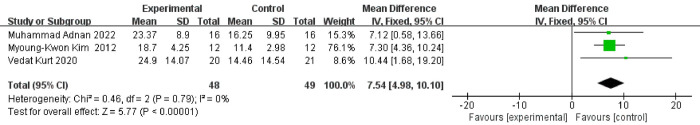
Comparison of ODI scores between experimental and control groups.

Three studies reported effects of NM on disability through the use of ODI [28, 30, 31]. The test of heterogeneity revealed the following results: χ^2^ = 0.46, I^2^ = 0%, P = 0.79, using a fixed effects model. The meta-analysis results indicated a mean difference (MD) of 7.54, with a 95% confidence interval (CI) of (4.98, 10.10), and a p-value less than 0.00001. These findings demonstrate statistical significance, suggesting that NM is superior to the control group in terms of improving ODI scores. **Fig 4**.

#### 2.4.3 Degree of straight leg raise

**Fig 5:**
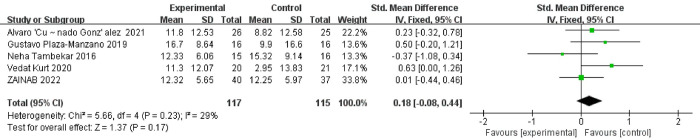
Comparison of degree of SLR between experimental and control groups.

Three studies have reported the effect of NM on SLR by using straight leg elevation after pain occurs at an angle [17, 29, 31–33]. The test for heterogeneity yielded the following results: χ^2^ = 5.66, I^2^ = 29%, P = 0.23, using a fixed effects model. The meta-analysis results indicated a mean difference (MD) of 0.18, with a 95% confidence interval (CI) of (-0.08, 0.44), and a p-value of 0.17. These results were not statistically significant, suggesting that there was no significant difference between NM and the control group in terms of the SLR improvement. **Fig 5**.

### 2.5 Subgroup analysis

**Fig 6:**
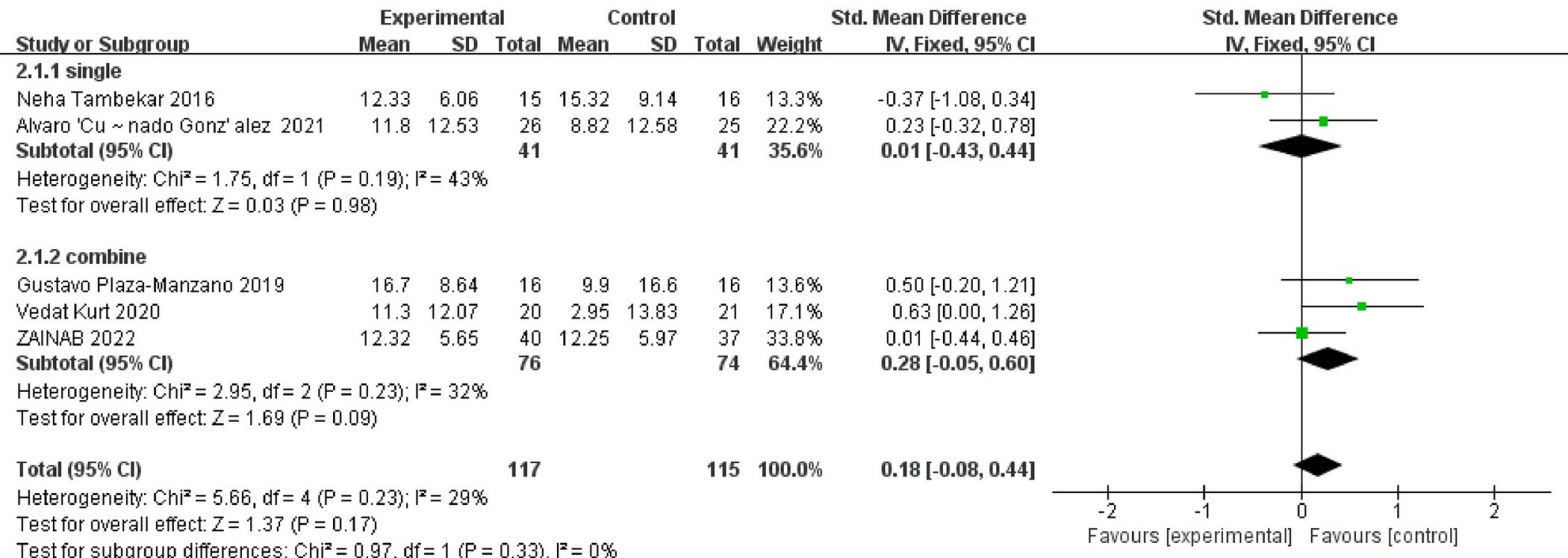
Comparison of SLR subgroup analysis between experimental and control groups. Note: Single indicates nerve fixation only, combined indicates nerve fixation in combination with other treatments.

Although the above experimental results concluded that there was no significant difference in SLR improvement between the NM technology group and the control group, the included literature showed that the combination of NM technology and other technologies improved the SLR level. Considering this problem and the reasons for the heterogeneity, a subgroup analysis was performed. Sources of heterogeneity in this study include diverse populations, interventions, publication bias, and study design and methodological quality. For example, the inconsistent duration and frequency of intervention, the inconsistent intervention means, and the inconsistent level of interveners may lead to certain bias in the outcome indicators of different literatures, thus affecting the results of this study. The meta-analytical outcomes revealed the following: Within the NM-only subgroup, the obtained p-value of 0.98 indicated a lack of statistical significance. This suggests an absence of notable disparity in the efficacy of NM as a standalone intervention regarding SLR improvement. Similarly, within the combined treatment subgroup, the computed p-value of 0.09 also pointed towards a lack of statistical significance. This implies a comparable efficacy between the combined treatment regimen and NM-only intervention concerning their impact on SLR improvement. The graphical representation of these findings is presented in **Fig 6.**

## 3 Discussion

### 3.1 Effect on pain

The meta-analysis of this study showed that the use of NM could effectively improve pain in patients with low back pain (P < 0.05). A study conducted by Kurt V et al. [31] supported this finding, demonstrating that the addition of NM to conventional treatment resulted in pain reduction and improved motor function among patients. Peacock M et al. [11] suggest that while NM is effective in reducing pain, its effects may be short-lived, and the long-term effectiveness remains uncertain. Several factors could potentially contribute to the variability in the long-term effectiveness of NM. These factors may include the strength and technique of the therapist’s manipulation, the type of manipulation employed, the duration of treatment, the patient’s tolerance, the duration of illness, and the severity of the disease. However, it is evident that NM plays a role in relieving nerve compression and improving nerve function, ultimately leading to the alleviation of symptoms [34]. Pain relief through NM may be attributed to the gate control theory. According to this theory, the activation of fine fibers (C-fibers) leads to the transmission of pain signals, while the activation of coarse fibers (A-β fibers) inhibits the activity of neurons, thereby reducing signal transmission and effectively closing the "gate" for pain information to reach the brain. This mechanism ultimately results in pain reduction [35–37].

NM distinctive slide and tension techniques have been shown to effectively enhance the flexibility of the musculoskeletal and peripheral nervous systems. These techniques can contribute to reducing the cross-sectional area of the nerves, minimizing peripheral and central sensory impairments. Furthermore, NM can help reduce the sensitivity of individuals with nervous system disorders to mechanical pressure and light touch [38]; NM can contribute to reducing neurogenic inflammation by diminishing the generation of anti-mechanical impulses in C fibers. By doing so, it reduces the input of sensory signals to the dorsal horn of the spinal cord [16]. When sensory information is insufficient, nerve impulses may not reach the pain threshold, resulting in the absence of pain sensation [36].

### 3.2 Effect on dysfunction and disability

This study found that NM could effectively reduce ODI scores [28, 30, 31], but it doesn’t necessarily improve quality of life. In a study conducted by Plaza Manzano et al. [32] on NM for lumbar spine disease, it was found that the addition of NM therapy to motor control did not result in a statistically significant change in patients’ function, as measured by the Rowland-Morris Disability Questionnaire (PMDQ), after a period of 2 months. As in the study of Kurt, V et al. [31] treatment with NM does not effectively improve the gait of patients, which is equivalent to failing to improve the disability level of patients. Dysfunction and its impact on quality of life are not always directly related. It is possible for dysfunction to occur without significantly affecting quality of life, or vice versa. NM, through its stimulation of nerves and soft tissues, promotion of blood circulation and material metabolism, and regulation of the nervous and immune systems, can improve the physical and psychological well-being of patients with LBP.

### 3.3 Effect on straight leg raise

The studies that participated in this subgroup analysis some suggest that NM could improve SLR in patients, but when these studies were included in the meta-analysis, results using NM alone (95%CI = -0.43 ~ 0.44, P = 0.98) were not found to improve SLR in patients and the results of using NM in combination with other treatments were contrary to the results of the investigators (95%CI = -0.05–0.60, P = 0.09), which may be the cause of insufficient sample size or large differences in sample size. Although a single study may show significant differences, when they are summarized, the combined results may no longer show significant differences due to heterogeneity, and there may be differences in the design, measurement methods and population characteristics of different studies due to methodological reasons. These differences may affect the consistency of the findings. Therefore, the effect of NM on SLR cannot be determined in this study, which needs to be further explored in prospective trials, although there are a few studies that suggest that NM is helpful in improving SLR. The SLR test is commonly utilized to evaluate the flexibility of the nerve structures in the lower limb [39]. The coordination and cooperation between the nervous system and the musculofemoral system play a crucial role in movement. The nervous system assists in generating muscle strength, while also influencing muscle balance and stability [40]. Interestingly, NM demonstrated comparable or even superior effectiveness in improving SLR levels when compared to other treatments. Three literature reports in the study [17, 29, 33], NM has been found to provide short-term improvements in SLR levels. However, its long-term efficacy is limited, and there is no significant advantage observed when combined with other treatment options. In a randomized controlled trial conducted by Kiran Satpute, clinically meaningful improvements were observed in SLR outcomes in the treatment of patients with radicular LBP. This improvement was achieved through two interventions over a 6-week period, involving spinal activity and leg exercises. Furthermore, the study demonstrated the long-term effects of these interventions [41]. The effectiveness of NM in improving outcomes may be attributed to the specific techniques used during treatment. Nerve Sliding can result in a bias of the nerve within adjacent tissues, while nerve tension may increase nerve pressure [42].

As mentioned above, although there are studies that believe that NM can improve the degree of SLR, there are also studies that believe that it is ineffective [31]. After analysis, this study found that the effectiveness is doubtful (P > 0.05), so it is not possible to determine its effectiveness at present, and more high-quality randomized controlled trial studies are needed to verify it in the future.

## 4 Limitations

There is a limited number of high-quality randomized controlled studies available on NM for the treatment of LBP, resulting in a smaller number of included literatures, this may compromise the reliability and generality of the results. Furthermore, some of the included literature may have a higher risk of bias. Additionally, most of the studies have small sample sizes and predominantly rely on subjective evaluation indicators such as scales, which may have reduced the statistical power of the experiments and increased the instability of the results. In the study of subjective evaluation indicators such as scale, there may be problems such as inconsistent evaluation criteria and subjective bias, which will affect the evaluation results. The lack of objective instruments and detailed experimental procedures is also notable, and some studies are not double-blind, which may introduce potential bias. Moreover, this study did not analyze factors such as safety, type of LBP, gender, and treatment duration. It is recommended that future researchers strive to enlarge the sample size to increase the representativeness and credibility of the study; Reduce the reliance on subjective evaluation and adopt objective quantitative methods as much as possible; At the same time, we advocate the pre-registration research plan, which is conducive to the repeatability of the experiment and improve the authenticity and reliability of the experiment.

## 5 Conclusions

NM demonstrated effectiveness in improving VAS and ODI outcomes in patients with LBP, but SLR outcomes are still uncertain and until more high-quality studies are included, the effectiveness of NM for SLR remains unknown.

## Supporting information

S1 Checklist Search formula. Note: PubMed search formula.
(PDF)

S1 Fig (TIF)

## References

[1] Coulombe BJ, Games KE, Neil ER, Eberman LE. Core Stability Exercise Versus General Exercise for Chronic Low Back Pain. J Athl Train. 2017;52(1):71–2. Epub 2016/11/17. doi: 10.4085/1062-6050-51.11.16 ; PubMed Central PMCID: PMC5293521. 27849389 PMC5293521

[2] Hayden JA, Ellis J, Ogilvie R, Malmivaara A, van Tulder MW. Exercise therapy for chronic low back pain. Cochrane Database Syst Rev. 2021;9(9):CD009790. Epub 2021/09/29. doi: 10.1002/14651858.CD009790.pub2 ; PubMed Central PMCID: PMC8477273. 34580864 PMC8477273

[3] Hauser RA, Matias D, Woznica D, Rawlings B, Woldin BA. Lumbar instability as an etiology of low back pain and its treatment by prolotherapy: A review. J Back Musculoskelet Rehabil. 2022;35(4):701–12. Epub 2021/12/28. doi: 10.3233/BMR-210097 ; PubMed Central PMCID: PMC9398090. 34957989 PMC9398090

[4] Barrey CY, Le Huec JC, French Society for Spine S. Chronic low back pain: Relevance of a new classification based on the injury pattern. Orthop Traumatol Surg Res. 2019;105(2):339–46. Epub 2019/02/23. doi: 10.1016/j.otsr.2018.11.021 . 30792166

[5] Corp N, Mansell G, Stynes S, Wynne-Jones G, Morso L, Hill JC, et al. Evidence-based treatment recommendations for neck and low back pain across Europe: A systematic review of guidelines. Eur J Pain. 2021;25(2):275–95. Epub 2020/10/17. doi: 10.1002/ejp.1679 ; PubMed Central PMCID: PMC7839780. 33064878 PMC7839780

[6] Pangarkar SS, Kang DG, Sandbrink F, Bevevino A, Tillisch K, Konitzer L, et al. VA/DoD Clinical Practice Guideline: Diagnosis and Treatment of Low Back Pain. J Gen Intern Med. 2019;34(11):2620–9. Epub 2019/09/19. doi: 10.1007/s11606-019-05086-4 ; PubMed Central PMCID: PMC6848394. 31529375 PMC6848394

[7] Wang XQ, Wang YL, Witchalls J, Han J, Zhang ZJ, Page P, et al. Physical therapy for acute and sub-acute low back pain: A systematic review and expert consensus. Clin Rehabil. 2024:2692155241229398. Epub 2024/02/06. doi: 10.1177/02692155241229398 . 38317586

[8] Korownyk CS, Montgomery L, Young J, Moore S, Singer AG, MacDougall P, et al. PEER simplified chronic pain guideline: Management of chronic low back, osteoarthritic, and neuropathic pain in primary care. Can Fam Physician. 2022;68(3):179–90. Epub 2022/03/17. doi: 10.46747/cfp.6803179 ; PubMed Central PMCID: PMC9833192. 35292455 PMC9833192

[9] Bailly F, Trouvin AP, Bercier S, Dadoun S, Deneuville JP, Faguer R, et al. Clinical guidelines and care pathway for management of low back pain with or without radicular pain. Joint Bone Spine. 2021;88(6):105227. Epub 2021/05/30. doi: 10.1016/j.jbspin.2021.105227 . 34051387

[10] Knezevic NN, Candido KD, Vlaeyen JWS, Van Zundert J, Cohen SP. Low back pain. Lancet. 2021;398(10294):78–92. Epub 2021/06/12. doi: 10.1016/S0140-6736(21)00733-9 . 34115979

[11] Peacock M, Douglas S, Nair P. Neural mobilization in low back and radicular pain: a systematic review. J Man Manip Ther. 2023;31(1):4–12. Epub 2022/05/19. doi: 10.1080/10669817.2022.2065599 ; PubMed Central PMCID: PMC9848316. 35583521 PMC9848316

[12] Basson A, Olivier B, Ellis R, Coppieters M, Stewart A, Mudzi W. The Effectiveness of Neural Mobilization for Neuromusculoskeletal Conditions: A Systematic Review and Meta-analysis. J Orthop Sports Phys Ther. 2017;47(9):593–615. Epub 2017/07/14. doi: 10.2519/jospt.2017.7117 . 28704626

[13] Paquette P, Lamontagne M, Higgins J, Gagnon DH. Repeatability and Minimal Detectable Change in Longitudinal Median Nerve Excursion Measures During Upper Limb Neurodynamic Techniques in a Mixed Population: A Pilot Study Using Musculoskeletal Ultrasound Imaging. Ultrasound Med Biol. 2015;41(7):2082–6. Epub 2015/04/15. doi: 10.1016/j.ultrasmedbio.2015.03.015 . 25868536

[14] Ijaz MJ, Karimi H, Ahmad A, Gillani SA, Anwar N, Chaudhary MA. Comparative Efficacy of Routine Physical Therapy with and without Neuromobilization in the Treatment of Patients with Mild to Moderate Carpal Tunnel Syndrome. Biomed Res Int. 2022;2022:2155765. Epub 2022/07/06. doi: 10.1155/2022/2155765 ; PubMed Central PMCID: PMC9242805 competing interests to report. 35782066 PMC9242805

[15] Dwornik M, Kujawa J, Bialoszewski D, Slupik A, Kiebzak W. Electromyographic and clinical evaluation of the efficacy of neuromobilization in patients with low back pain. Ortop Traumatol Rehabil. 2009;11(2):164–76. Epub 2009/06/09. . 19502673

[16] Alshami AM, Alghamdi MA, Abdelsalam MS. Effect of Neural Mobilization Exercises in Patients With Low Back-Related Leg Pain With Peripheral Nerve Sensitization: A Prospective, Controlled Trial. J Chiropr Med. 2021;20(2):59–69. Epub 2022/01/07. doi: 10.1016/j.jcm.2021.07.001 ; PubMed Central PMCID: PMC8703155. 34987322 PMC8703155

[17] Tambekar N, Sabnis S, Phadke A, Bedekar N. Effect of Butler’s neural tissue mobilization and Mulligan’s bent leg raise on pain and straight leg raise in patients of low back ache. J Bodyw Mov Ther. 2016;20(2):280–5. Epub 2016/05/24. doi: 10.1016/j.jbmt.2015.08.003 . 27210844

[18] Wu J, Zeng A, Chen Z, Wei Y, Huang K, Chen J, et al. Effects of Virtual Reality Training on Upper Limb Function and Balance in Stroke Patients: Systematic Review and Meta-Meta-Analysis. J Med Internet Res. 2021;23(10):e31051. Epub 2021/10/13. doi: 10.2196/31051 ; PubMed Central PMCID: PMC8548971. 34636735 PMC8548971

[19] Khodadad B, Letafatkar A, Hadadnezhad M, Shojaedin S. Comparing the Effectiveness of Cognitive Functional Treatment and Lumbar Stabilization Treatment on Pain and Movement Control in Patients With Low Back Pain. Sports Health. 2020;12(3):289–95. Epub 2019/12/17. doi: 10.1177/1941738119886854 ; PubMed Central PMCID: PMC7222662. 31841078 PMC7222662

[20] Bryndal A, Majchrzycki M, Grochulska A, Glowinski S, Seremak-Mrozikiewicz A. Risk Factors Associated with Low Back Pain among A Group of 1510 Pregnant Women. J Pers Med. 2020;10(2). Epub 2020/06/19. doi: 10.3390/jpm10020051 ; PubMed Central PMCID: PMC7354496. 32549306 PMC7354496

[21] Zhong Y, Liu J, Zhou W, Yu D. Relationship between straight leg-raising test measurements and area of fat infiltration in multifidus muscles in patients with lumbar disc hernation. J Back Musculoskelet Rehabil. 2020;33(1):57–63. Epub 2019/04/23. doi: 10.3233/BMR-181304 . 31006661

[22] Gross A, Kay TM, Paquin JP, Blanchette S, Lalonde P, Christie T, et al. Exercises for mechanical neck disorders. Cochrane Database Syst Rev. 2015;1(1):CD004250. Epub 2015/01/30. doi: 10.1002/14651858.CD004250.pub5 ; PubMed Central PMCID: PMC9508492. 25629215 PMC9508492

[23] Hu X, Ma M, Zhao X, Sun W, Liu Y, Zheng Z, et al. Effects of exercise therapy for pregnancy-related low back pain and pelvic pain: A protocol for systematic review and meta-analysis. Medicine (Baltimore). 2020;99(3):e17318. Epub 2020/02/06. doi: 10.1097/MD.0000000000017318 ; PubMed Central PMCID: PMC7220333. 32011431 PMC7220333

[24] Hu HT, Gao H, Ma RJ, Zhao XF, Tian HF, Li L. Is dry needling effective for low back pain?: A systematic review and PRISMA-compliant meta-analysis. Medicine (Baltimore). 2018;97(26):e11225. Epub 2018/06/29. doi: 10.1097/MD.0000000000011225 ; PubMed Central PMCID: PMC6242300. 29952980 PMC6242300

[25] Higgins JPT, Thomas J, Chandler J, Cumpston M, Li T, Page MJ, Welch VA (editors). Cochrane Handbook for Systematic Reviews of Interventions. 2nd Edition. Chichester (UK): John Wiley & Sons, 2019. Available from: https://handbook-5-1.cochrane.org/chapter_16/16_1_3_2_imputing_standard_deviations_for_changes_from_baseline.htm

[26] Tufanaru C, Munn Z, Stephenson M, Aromataris E. Fixed or random effects meta-analysis? Common methodological issues in systematic reviews of effectiveness. International journal of evidence-based healthcare. 2015;13(3):196–207. doi: 10.1097/XEB.0000000000000065 MEDLINE: 26355603. 26355603

[27] Wang P, Zuo G, Du SQ, Gao TC, Liu RJ, Hou XZ, et al. Meta-analysis of the therapeutic effect of acupuncture and chiropractic on cervical spondylosis radiculopathy: A systematic review and meta-analysis protocol. Medicine (Baltimore). 2020;99(5):e18851. Epub 2020/02/01. doi: 10.1097/MD.0000000000018851 ; PubMed Central PMCID: PMC7004792. 32000386 PMC7004792

[28] Adnan M, Arsh A, Ali B, Ahmad S. Effectiveness of bent leg raise technique and neurodynamics in patients with radiating low back pain. Pak J Med Sci. 2022;38(1):47–51. Epub 2022/01/18. doi: 10.12669/pjms.38.1.4010 ; PubMed Central PMCID: PMC8713242. 35035399 PMC8713242

[29] Gonzalez AC, Berenguer SB, Manas JML, Martin-Pintado-Zugasti A. Validation of a sham novel neural mobilization technique in patients with non-specific low back pain: A randomized, placebo-controlled trial. Musculoskeletal Science and Practice. 2021;53. doi: 10.1016/j.msksp.2021.102378 WOS:000656433800014. 33930856

[30] Kim M-K, Ji S-G, Cha H-K, Chang J-S. Effects of Electromagnetic Diathermy in Conjunction with Nerve Mobilization in the Management of Lower Back Pain. Journal of Physical Therapy Science. 2012;24(12):1337–9. doi: 10.1589/jpts.24.1337 WOS:000320683800028.

[31] Kurt V, Aras O, Buker N. Comparison of conservative treatment with and without neural mobilization for patients with low back pain: A prospective, randomized clinical trial. J Back Musculoskelet Rehabil. 2020;33(6):969–75. Epub 2020/03/08. doi: 10.3233/BMR-181241 . 32144973

[32] Plaza-Manzano G, Cancela-Cilleruelo I, Fernandez-de-las-Penas C, Cleland JA, Arias-Buria JL, Thoomes-de-Graaf M, et al. Effects of Adding a Neurodynamic Mobilization to Motor Control Training in Patients With Lumbar Radiculopathy Due to Disc Herniation A Randomized Clinical Trial. American Journal of Physical Medicine & Rehabilitation. 2020;99(2):124–32. doi: 10.1097/PHM.0000000000001295 WOS:000507925800010. 31464753

[33] Zainab, Anwar S, Avaid A, Fatimah W, Perveen W, Naseem N. Effects of Sciatic Nerve Mobilization on Pain, Disability and Range in Patients with Lumbar Radicular Pain. Pakistan journal of medical and health sciences. 2022;16(10):97‐9. doi: 10.53350/pjmhs22161097 CN-02503473.

[34] Ahmad Siraj S, Dadgal R. Physiotherapy for Piriformis Syndrome Using Sciatic Nerve Mobilization and Piriformis Release. Cureus. 2022;14(12):e32952. Epub 2023/01/31. doi: 10.7759/cureus.32952 ; PubMed Central PMCID: PMC9879580. 36712711 PMC9879580

[35] Lin T, Gargya A, Singh H, Sivanesan E, Gulati A. Mechanism of Peripheral Nerve Stimulation in Chronic Pain. Pain Med. 2020;21(Suppl 1):S6–S12. Epub 2020/08/18. doi: 10.1093/pm/pnaa164 ; PubMed Central PMCID: PMC7828608. 32804230 PMC7828608

[36] Chen Q, Wang Z, Zhang S. Mechanism, application and effect evaluation of nerve mobilization in the treatment of low back pain: A narrative review. Medicine (Baltimore). 2023;102(34):e34961. Epub 2023/09/01. doi: 10.1097/MD.0000000000034961 ; PubMed Central PMCID: PMC10470699. 37653794 PMC10470699

[37] Mendell LM. Constructing and deconstructing the gate theory of pain. Pain. 2014;155(2):210–6. Epub 2013/12/18. doi: 10.1016/j.pain.2013.12.010 ; PubMed Central PMCID: PMC4009371. 24334188 PMC4009371

[38] Gonzalez-Matilla R, Abuin-Porras V, Casuso-Holgado MJ, Riquelme I, Heredia-Rizo AM. Effects of neural mobilization in disorders associated with chronic secondary musculoskeletal pain: A systematic review and meta-analysis. Complement Ther Clin Pract. 2022;49:101618. Epub 2022/07/04. doi: 10.1016/j.ctcp.2022.101618 . 35780543

[39] Stepien A, Paldyna B. Neurodynamic Functions and Their Correlations with Postural Parameters in Adolescents with Idiopathic Scoliosis. J Clin Med. 2022;11(4). Epub 2022/02/26. doi: 10.3390/jcm11041115 ; PubMed Central PMCID: PMC8880101. 35207387 PMC8880101

[40] Azzollini V, Dalise S, Chisari C. How Does Stroke Affect Skeletal Muscle? State of the Art and Rehabilitation Perspective. Front Neurol. 2021;12:797559. Epub 2022/01/11. doi: 10.3389/fneur.2021.797559 ; PubMed Central PMCID: PMC8733480. 35002937 PMC8733480

[41] Satpute K, Hall T, Bisen R, Lokhande P. The Effect of Spinal Mobilization With Leg Movement in Patients With Lumbar Radiculopathy-A Double-Blind Randomized Controlled Trial. Arch Phys Med Rehabil. 2019;100(5):828–36. Epub 2018/12/07. doi: 10.1016/j.apmr.2018.11.004 . 30521781

[42] Martins C, Pereira R, Fernandes I, Martins J, Lopes T, Ramos L, et al. Neural gliding and neural tensioning differently impact flexibility, heat and pressure pain thresholds in asymptomatic subjects: A randomized, parallel and double-blind study. Phys Ther Sport. 2019;36:101–9. Epub 2019/02/03. doi: 10.1016/j.ptsp.2019.01.008 . 30710858

