# Supplementary figures and images for: Efficacy of neuromobilization in the treatment of low back pain: Systematic review and meta-analysis
Source: PLoS One. 2024 May 7;19(5):e0302930. doi: 10.1371/journal.pone.0302930 (PMC11075829; doi:10.1371/journal.pone.0302930)

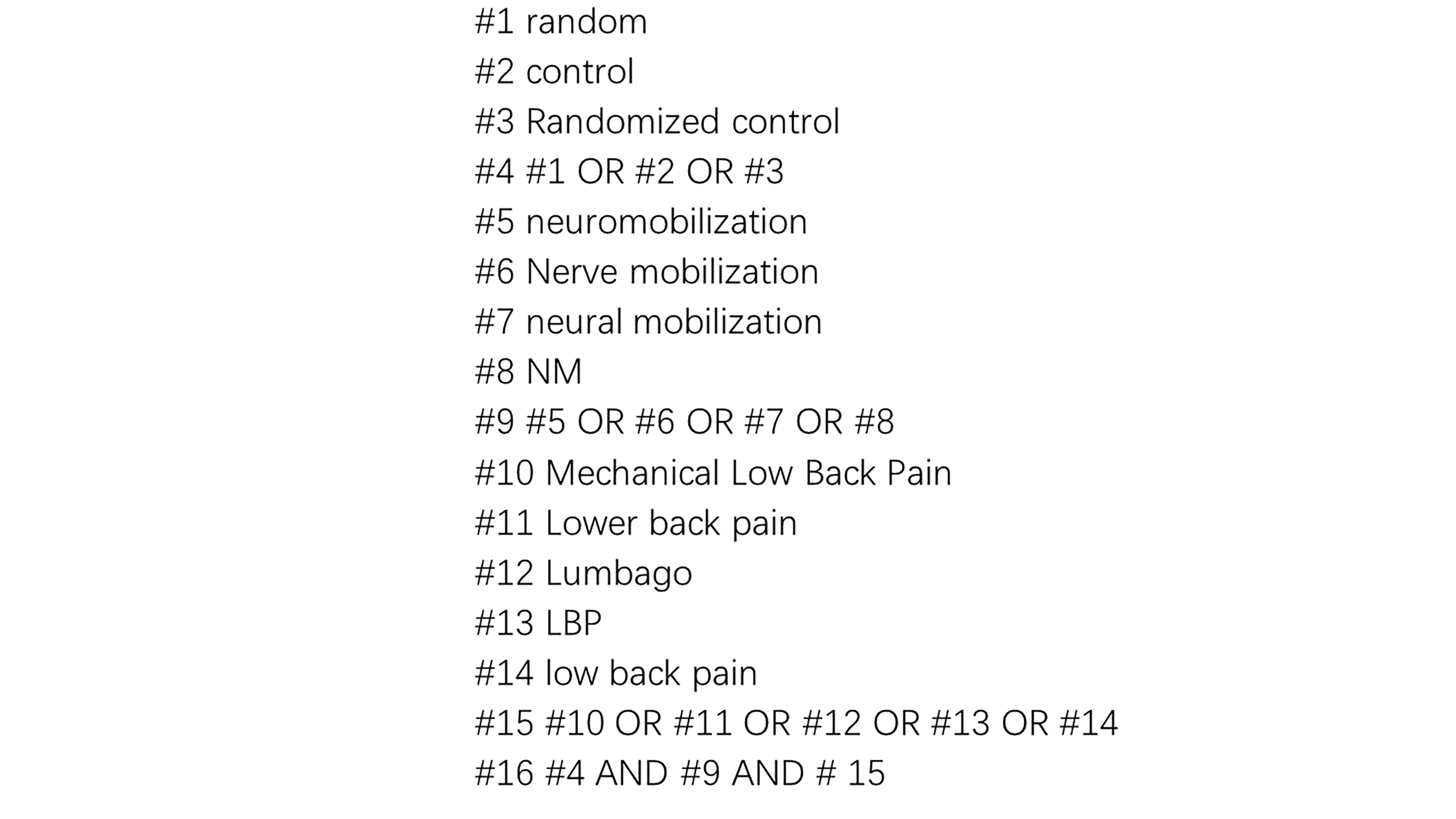

Supplement: S1 Fig — (TIF) [file pone.0302930.s002.tif]
